# Case Report: Portal cavernoma secondary to multiple liver hydatidosis: A rare cause of cataclysmic haemorrhage in a young adult

**DOI:** 10.12688/f1000research.74012.1

**Published:** 2021-10-29

**Authors:** Alia Zouaghi, Nawel Bellil, Khalaf Ben Abdallah, Dhafer Hadded, Haithem Zaafouri, Mona Cherif, Anis Ben Maamer

**Affiliations:** 1 Department of General Surgery, Habib Thameur Hospital, Tunis, 1008, Tunisia; 2 Department of Gastroenterology, Habib Thameur Hospital, Tunis, 1008, Tunisia

**Keywords:** Case report, portal cavernoma, variceal bleeding, hydatidosis

## Abstract

Clinical presentation of liver hydatidosis can vary from asymptomatic forms to lethal complications. We report a rare case of a 27-year-old male from a rural Tunisian region who presented with large-abundance haematemesis, haemodynamic instability, and marked biological signs of hypersplenism. Endoscopy showed bleeding esophageal varicose veins that were ligated. Abdominal ultrasound concluded the presence of three type CE2 hydatic liver cysts causing portal cavernoma with signs of portal hypertension. Despite resuscitation, the patient died of massive rebleeding leading to haemorrhagic shock. Hepatic hydatid cyst should be considered as an indirect cause of gastrointestinal bleeding in endemic countries. Early abdominal ultrasound in varicose haemorrhage is essential in orienting the diagnosis.

## Introduction

Echinococcosis liver hydatidosis is endemic in the Maghreb countries. Vascular complications are exceedingly rare, ^1^ however, its manifestations can be critical. The clinical presentation depends on the cyst’s segment localization. Compression or invasion of the hydatic cyst in the portal vein can lead to portal vein thrombosis or extrahepatic portal vein obstruction (EPVHO). ^2^ This can rarely lead to cavernous transformation and portal hypertension explaining the origin of symptoms. Prognosis is generally good. Cataclysmic presentations have not been described in the literature.

## Case presentation

A 27-year-old male patient, from a rural area of Tunisia, without any medical history, presented to the Habib Thameur Hospital emergency room with massive upper gastrointestinal bleeding. On admission (day one) in August 2020, physical examination revealed diffuse mucocutaneous pallor, lesions of old scarifications in the left upper limb, a Glasgow Coma Score (GCS) of 15/15, tachycardia of 105 bpm, hypotension of 80/50 mmHg without signs of peripheral hypoperfusion. Abdominal examination revealed slight epigastric tenderness and splenomegaly without hepatomegaly or skin signs of hepatocellular failure. The rectal digital examination came back stained with melena.

Laboratory investigation showed signs of hypersplenism including decreased count of white blood count of 2870 cells/mm ^3^, thrombocytopenia of 46,000 cells/mm ^3^, and normochromic normocytic anemia of 5.6 g/dL. Minor signs of hepatocellular insufficiency were also displayed including a low rate of prothrombin ratio of 60% and hypocholesterolemia of 2.87 mmol/L. He had neither cholestasis nor cytolysis. Albuminemia was normal at the value of 36 g/L. Acute kidney failure was noted (urea of 11.6 mmol/L) with a normal blood ionogram. Viral hepatitis screening was negative.

**Figure 1.**
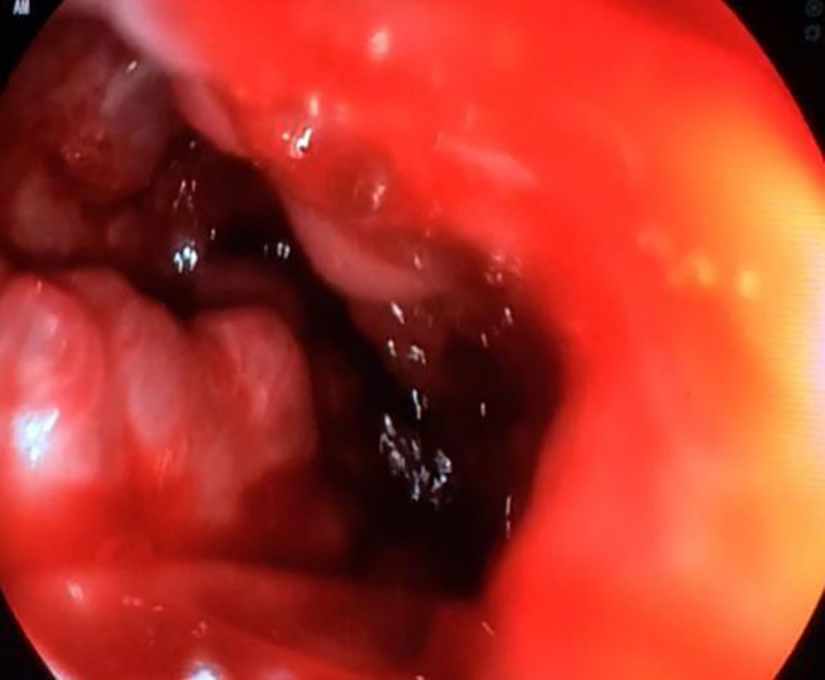
An upper gastroduodenoscopy demonstrating the presence of esophageal varices complicated by massive active bleeding, and gastric varicose veins.

On day two, the patient was stabilized following fluid resuscitation and blood transfusion of two red blood cell concentrates. He was put on a proton-pump inhibitor (omeprazole) and octreotide. Upper gastroduodenoscopy showed the presence of oesophageal varices with massive active bleeding, moderate hypertensive gastropathy and gastric varicose veins ( Figure 1). Four elastics with a first kit of ligature were put in place but did not allow the control of bleeding. Five supplementary elastics in a second ligature kit allowed a reduction in bleeding but without total control of the haemorrhage.

**Figure 2.**
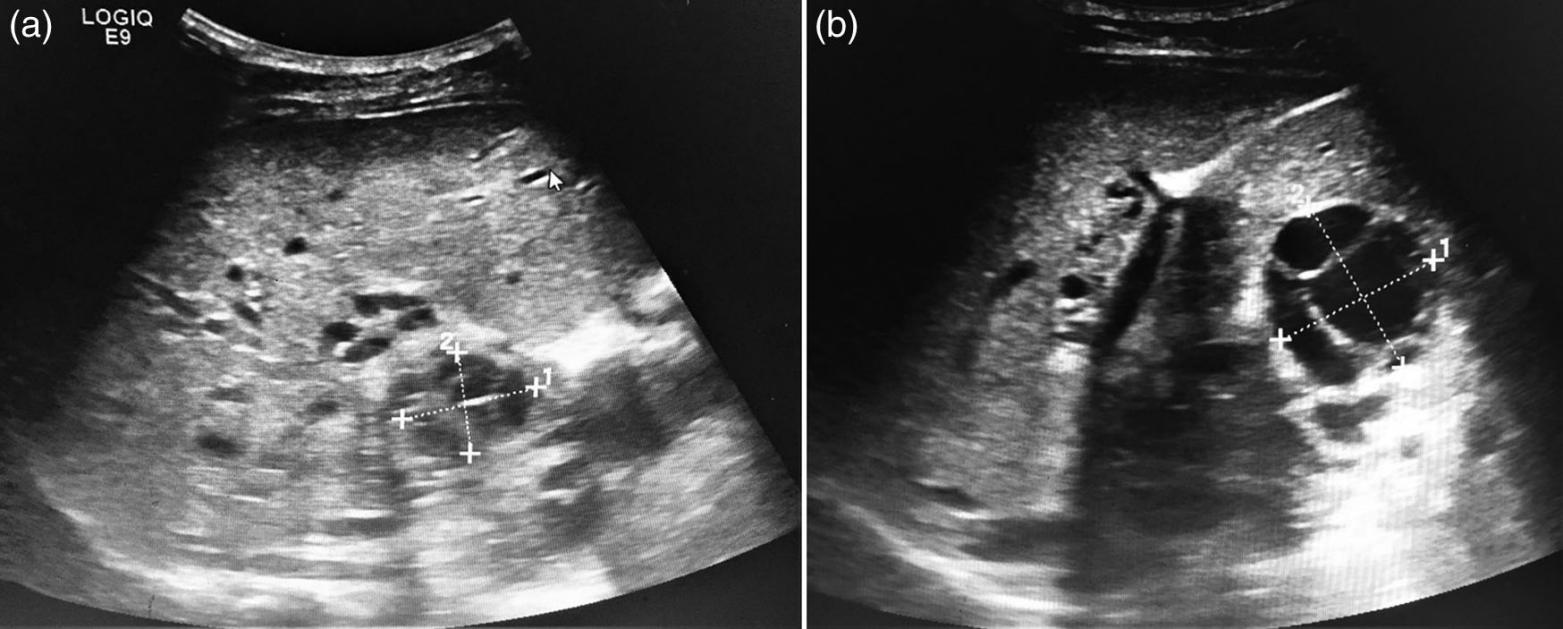
a and b: An abdominal ultrasound showing the presence of multiple multiloculated cystic formations evoking hydatic cysts type CE2 of the WHO classification of segments I, IV, and V.

An abdominal ultrasound concluded the presence of multiple multiloculated cystic formations evoking hydatic cysts type CE2 of the WHO classification of segments I, IV, and V with the largest cyst measuring 40 mm ( Figures 2a and 2b). A portal cavernoma with a dilated splenic vein and splenomegaly of 22 cm was described. Hepatic veins were permeable with a normal caliber.

After a week, the patient had a cataclysmic re-bleeding causing refractory hemorrhagic shock and disseminated intravenous coagulation leading to death.

## Discussion

Hepatic hydatid cyst can invade or compress the portal vein and results in EPVHO. Portal cavernoma is the outcome of chronic portal vein obstruction. It results in the creation of collateral circulation. ^4^

As soon as portal hypertension is established, blood can run hepatopetal or hepatofugal from the liver through portosystemic collaterals. This leads to the development of gastroesophageal or ectopic rectal variceal bleeding which represents the most typical symptomatic presentation as in our case. ^5^ According to a prospective study by Noronha *et al*., (2016), 71% of patients with chronic EPVHO showed gastroesophageal varices in endoscopy. ^5^

EPVHO is an uncommon entity in adults. Indeed, the incidence of having EPVHO for the general population does not exceed 1% with a very low mortality rate. ^6^ Hepatic hydatid cyst can results in EPVHO whether by compressing or directly invading the portal vein ^1^^,^^7^ Computerized tomography (CT) can help indicate the mechanism of thrombosis. Venous compressions are among the rarest complications of hydatid liver cysts even in endemic countries as shown in Table 1. Size and perivascular topography are two essential elements for its occurrence ( tunisiechirurgicale.com).

**Table 1.** Incidence of vascular complications in liver hydatidosis in the literature.

Series	Saleh *et al*., ^8^	Eddeghai *et al*., ^9^	Hafi *et al*., ^10^	Ben Ameur *et al*., [ tunisiechirurgicale.com]
Incidence	0.8 %	1 %	0.8%	1.8%

From a pathophysiological point of view, EPVHO is the consequence of the ‘Virchow’s Triad’: the extrinsic chronic compression of the vascular wall by the hydatic cysts decreases the blood flow rate thus creating endothelial injuries. This can lead to the formation of a bland thrombus.

To our knowledge, few cases of hepatic hydatidosis revealed by portal hypertension have been reported in the medical literature since the publication of the first case in 1990. ^8^^–^^10^ In a large Spanish cohort of 506 patients followed over 20 years, only two patients presented portal hypertension and variceal haemorrhage. ^11^ This demonstrates how seldomly described is this complication in the literature.

Our case offers an unusual presentation. The portal vein thrombosis and the cavernous transformation remained undiagnosed until hematemesis due to varices rupture. Our young patient had no history of inflammatory bowel diseases, pancreatitis, cirrhosis, neoplastic condition or coagulopathy to explain the cavernoma. The only convincing cause was the compression of the hydatid cysts respectively in segments I, IV, and V of the portal vein leading to chronic thrombosis. The minor biological signs of hepatocellular insufficiency were probably secondary to the chronic evolution of the cavernoma, but the ultrasound did not show any signs of chronic hepatopathy. Ultrasound was an indispensable tool to orienting diagnosis as proved in the literature. ^12^ In this particular case, our patient's prognosis was poor, unlike previous ones.

We summarize in Table 2 different cases of hepatic hydatid cyst causing portal cavernoma. The mean age was 58.2 years old. No signs of gastrointestinal bleeding were reported before diagnosis. Portal thromboses were mainly secondary to direct compression or invasion of the portal vein. The outcome was generally positive in all cases unlike ours. Treatment was generally based on surgery and the drug albendazole.

**Table 2.** Cases of hepatic hydatid cyst causing portal cavernoma.

Case	Age	Clinical presentation	Endoscopy	Mechanism of thrombosis	Prognosis	Treatment
**Ertan *et al*.,** ^13^	77	Fever and generalized weakness	Not done	Direct communication with the portal vein	On follow-up	Albendazole
**Kirmizi *et al*.,** ^14^	33	Abdominal distension after meals	Oesophageal and gastric fundal varices with no bleeding	Compression of the hepatic hilum	Preserved	Surgery
**Moisan *et al*.,** ^15^	62	Abdominal pain Hepatomegaly	Grade I oesophageal varices	Invasion of the portal vein by daughter vesicles	Preserved	Albendazole + Propranolol
**Colovic *et al*.,** ^16^	56	Right upper quadrant abdominal pain	Normal	Compression of the hepatoduodenal ligament	Preserved	Surgery
**Kayecetin *et al*.,** ^17^	63	Fever, anorexia, jaundice and epigastric mass	Not done	Abdominal sepsis after the rupture of the liver cyst	Preserved	Surgery, Albendazole

Our case illustrates the importance of considering non-cirrhotic portal hypertension secondary to liver hydatidosis especially in young patients in endemic countries. Urgent therapy and diagnostic investigations should be pursued simultaneously to ensure on-time intervention. Our case lacks abdominal computed tomography or magnetic resonance imaging which could have given a more accurate demonstration of the thrombosis mechanism. Our patient could have been a candidate for transjugular intrahepatic portosystemic shunt.

## Conclusion

Liver hydatidosis remains a public health issue in Tunisia because of its high morbidity and, exceptionally, possible mortality. Our case highlights an unusual presentation due to the young age, symptomatic portal cavernoma, and unfortunate death of the patient. It shows the importance of considering hepatic hydatid cyst as a cause of portal hypertension and portal cavernoma on presentation of gastrointestinal bleeding. Early abdominal ultrasound has a valuable contribution to orienting diagnosis, especially in endemic countries.

## Data availability

All data underlying the results are available as part of the article and no additional source data are required.

## Consent

Written informed consent for publication of their clinical details and clinical images was obtained from the patient’s family.
