# Supplementary material for: Case Report: Portal cavernoma related to multiple liver hydatidosis: A rare case of fatal cataclysmic haemorrhage
Source: F1000Res. 2021 Nov 25;10:1097. Originally published 2021 Oct 29. [Version 2] doi: 10.12688/f1000research.74012.2 (PMC8630548; doi:10.12688/f1000research.74012.2)
Supplement: Peer review history

# Peer review: Reviewer response for version 2

**DOI:** 10.5256/f1000research.79569.r101158

**Published:** 2021-11-26

**Authors:** Souheil Zayet

**Affiliations:** 1 Infectious Disease Department, Nord Franche-Comté Hospital, Trévenans, France

The authors responded to all reviewer's comments.

Are enough details provided of any physical examination and diagnostic tests, treatment given and outcomes?

Yes

Is the case presented with sufficient detail to be useful for other practitioners?

Yes

Is sufficient discussion included of the importance of the findings and their relevance to future understanding of disease processes, diagnosis or treatment?

Partly

Is the background of the case’s history and progression described in sufficient detail?

Yes

Reviewer Expertise:

Specialist in Infectious Diseases

I confirm that I have read this submission and believe that I have an appropriate level of expertise to confirm that it is of an acceptable scientific standard.

# Peer review: Reviewer response for version 1

**DOI:** 10.5256/f1000research.77717.r98517

**Published:** 2021-11-16

**Authors:** Khaoula Chabbouh

**Affiliations:** 1 Gastroenterology B department, Faculty of Medicine, La Rabta Hospital, University Tunis-El-Manar, Tunis, Tunisia

This is an interesting case as it illustrates a rare complication of Echinococcosis liver hydatidosis which is still endemic in the Maghreb countries.

 However, some remarks should be mentioned:

1. The diagnosis was based on abdominal ultrasound findings only. Authors did not perform abdominal computed tomography or magnetic resonance imaging.
2. Given the young age of the patient, a thrombophilia assessment could have been requested.

Are enough details provided of any physical examination and diagnostic tests, treatment given and outcomes?

Yes

Is the case presented with sufficient detail to be useful for other practitioners?

Yes

Is sufficient discussion included of the importance of the findings and their relevance to future understanding of disease processes, diagnosis or treatment?

Yes

Is the background of the case’s history and progression described in sufficient detail?

Yes

Reviewer Expertise:

Gastro-enterology

I confirm that I have read this submission and believe that I have an appropriate level of expertise to confirm that it is of an acceptable scientific standard.

# Peer review: Reviewer response for version 1

**DOI:** 10.5256/f1000research.77717.r98522

**Published:** 2021-11-03

**Authors:** Souheil Zayet

**Affiliations:** 1 Infectious Disease Department, Nord Franche-Comté Hospital, Trévenans, France

**This submission is an observation of a patient with a rare presentation of portal cavernoma related to multiple liver hydatidosis.**

 This original document is well treated in its entirety and is of academic and scientific interest, although limited due to the incidence of the disease, but both necessary and useful to understand the infectious mechanisms and the care options. Personally, I find very enriching the approach of the authors and the way they described this case, associated with quality references.

 I believe the content is substantially acceptable. However as the article is intended for surgery/infectious diseases specialists and needed some minor revisions:

In the title:

**‘Portal cavernoma related to multiple liver hydatidosis: A rare case of fatal cataclysmic haemorrhage’ **is more appropriate.

In the Abstract:

 Correct ‘We report herein a rare case of a 27-year-old Tunisian male a rural region’.

 ‘Biological data’ instead signs.

In the Introduction:

 Add a reference with the Echinococcosis liver hydatidosis’ prevalence in the Maghreb countries.

 Please check the English of this sentence ‘Prognosis is generally good’. Please avoid jargon and relaxed language.

In the Observation:

 You can define gastrointestinal as ‘GI’ and used it in the whole text.

 Please precise the native region ‘rural area’ of patient to emphasize epidemiological link.

 Delete the name of your affiliation or institution in the text.

 Correct beats per minute; avoid abbreviations.

 Use ‘enlarged spleen’ instead splenomegaly.

 Define all normal ranges in laboratory investigations in the text.

 Replaced normal ionogram with ‘normal electrolytes’.

 What is ‘Viral hepatitis screening’; it means serology; if yes, precise all serology and rt-PCR included.

 He was put on a proton-pump inhibitor?? Please changed it (the patient was treated or a treatment was started/began).

 To retain the diagnosis, hydatidosis serology (or other microbiological testing) was done ? If not, precise it and discuss this point in the discussion part).

In the Discussion:

 Replace symptomatic presentation with ‘clinical feature’.

 Replace ‘offer’ with highlighted.

 Deleted CT as abbreviation, used once in the text.

 Please add a reference when you explain pathophysiology of EPVHO.

 Please check this sentence ‘The outcome was generally positive in all cases unlike ours’.

 Surgery technique was not detailed; (you can do it in 1-2 sentences.

In the conclusion:

 Avoid unfortunate; change it (lethal or fatal form). 

 Add ‘in endemic low-middle income countries’.

Are enough details provided of any physical examination and diagnostic tests, treatment given and outcomes?

Yes

Is the case presented with sufficient detail to be useful for other practitioners?

Yes

Is sufficient discussion included of the importance of the findings and their relevance to future understanding of disease processes, diagnosis or treatment?

Partly

Is the background of the case’s history and progression described in sufficient detail?

Yes

Reviewer Expertise:

Specialist in Infectious Diseases

I confirm that I have read this submission and believe that I have an appropriate level of expertise to confirm that it is of an acceptable scientific standard, however I have significant reservations, as outlined above.
